# Genome Analysis and Characterization of *Formosa bonchosmolovskayae* sp. nov. Isolated from Brown and Green Algae, and a Proposal to Reclassify *Formosa maritima* Cao et al. 2020 and *Bizionia arctica* Li et al. 2015 as *Xanthomarina* New Members

**DOI:** 10.3390/microorganisms14020328

**Published:** 2026-01-30

**Authors:** Olga Nedashkovskaya, Evgeniya Bystritskaya, Yulia Savicheva, Yulia Bronnikova, Nadezhda Otstavnykh, Viacheslav Eremeev, Song-Gun Kim, Natalia Zhukova, Marina Isaeva

**Affiliations:** 1 G.B. Elyakov Pacific Institute of Bioorganic Chemistry, Far Eastern Branch, Russian Academy of Sciences, Prospect 100 Let Vladivostoku, 159, Vladivostok 690022, Russia; ep.bystritskaya@yandex.ru (E.B.); iu.savicheva0@yandex.ru (Y.S.); molonova.yulya@gmail.com (Y.B.); chernysheva.nadezhda@gmail.com (N.O.); wieremeew@gmail.com (V.E.); 2 Korean Collection for Type Cultures, Biological Resource Center, Korea Research Institute of Bioscience and Biotechnology, 181 Ipsin-gil, Jeongeup-si 56212, Republic of Korea; sgkim@kribb.re.kr; 3 A.V. Zhirmunsky National Scientific Center of Marine Biology, Far Eastern Branch, Russian Academy of Sciences, Palchevskogo Street 17, Vladivostok 690041, Russia; nzhukova35@list.ru

**Keywords:** *Formosa bonchosmolovskayae*, *Flavobacteriaceae*, marine bacteria, brown alga *Saccharina japonica*, green alga *Ulva fenestrata*, whole genome sequence

## Abstract

Two marine bacteria, designated strains 4Alg 33^T^and 3Alg 14/1, were isolated from brown (*Saccharina japonica*) and green (*Ulva fenestrata*) macroalgae, respectively. These isolates were aerobic Gram-negative rods exhibiting a gliding motility. The 16S rRNA gene phylogenetic analysis clearly showed their belonging to the genus *Formosa*, the family *Flavobacteriaceae*, and the phylum *Bacteroidota*. The closest relatives of the new strains were *Formosa undariae* KCTC 32328^T^ (99.05%), *Formosa arctica* IMCC 9485^T^ (99.05%) and *Formosa agariphila* KMM 3901^T^ (98.96%). The ANI and dDDH values between the two new strains were 97.9% and 85.3%, respectively. The AAI values between 4Alg 33^T^ and *Formosa* type strains ranged from 80.1% (*Formosa haliotis* MA1^T^) to 91.4% (*F. undariae* KCTC 32328^T^). The cellular fatty acid and polar lipid profiles of the new isolates were generally similar to those of the type strains of *Formosa* species. The genomes of 4Alg 33^T^ and 3Alg 14/1 are represented by a circular chromosome of 4,157,724 bp and 4,316,096 bp in size with 3536 and 3879 protein-coding genes, respectively. They shared a DNA G+C content of 34.3 mol% and comprised four *rrn* operons. The pangenome of the genus *Formosa* belongs to the open type and is characterized by an abundance of CAZymes. The proportion of CAZyme genes in novel genomes was more than 5%, with a prevalence of glycoside hydrolase genes, suggesting great potential for utilizing marine-derived polysaccharides. Based on the results of polyphasic characterization, the two algal isolates represent a distinct species lineage within the genus *Formosa*, for which we propose the name *Formosa bonchosmolovskayae* sp. nov. with the type strain 4Alg 33^T^ (= KMM 3963^T^ = KCTC 72008^T^). In addition, we have proposed to transfer *Formosa maritima* Cao et al. 2020 and *Bizionia arctica* Li et al. 2015 to the genus *Xanthomarina* Vaidya et al. 2015 as *Xanthomarina maritima* comb. nov. and *Xanthomarina arctica* comb. nov. based on a combination of the genomic and phenotypic characteristics.

## 1. Introduction

The genus *Formosa*, a member of the family *Flavobacteriaceae* (phylum *Bacteroidota*)*,* was first identified by Ivanova et al. (2004) to denote Gram-negative, aerobic, rod-shaped and cytochrome-oxidase-negative marine bacteria isolated from an enrichment community degrading the brown alga *Fucus evanescens* [1]. Later, Nedashkovskaya et al. [2] amended this genus due to newly obtained phenotypic and genotypic data including the presence of gliding motility, budding fission, oxidase activity, phosphatidylethanolamine as the only phospholipid, and a DNA G+C content of 34–36 mol%. In 2015, Shakeela et al. [3] proposed a revised description of the genus based on the inability of *Formosa* species to utilize Tween 20 and the addition of aminolipids and lipids to their polar lipid profile. At last, based on the comparative phylogenomic analysis of the type strains of the phylum *Bacteroidota*, the genus *Formosa* was further emended by assigning a new DNA G+C content value of 31–40 mol% and adding a genome size parameter of 2.9–4.6 Mb [4].

To date, the genus *Formosa* includes seven species with a correct and validly published name (https://lpsn.dsmz.de/genus/formosa, accessed on 15 December 2025). All *Formosa* species have been isolated exclusively from marine habitats, most of them found in association with green and brown algae [1,2,5]. Other species were recovered from sediments and seawater [6,7,8]. One species was isolated from the abalone gut [9]. It is worth noting that a comprehensive genomic analysis of the type strain *Formosa agariphila* KMM 3901^T^ revealed a large number of genes encoding proteases and glycoside hydrolases, indicating a key role in polymer decomposition and strong specialization to the algal lifestyle [10].

During the research of the cultivable microbial diversity of green and brown algae, common in the coastal waters of the Sea of Japan, two aerobic, yellow-pigmented and gliding-motile bacterial strains were isolated. The new algal isolates were subsequently examined through a polyphasic taxonomic approach, which confirmed their classification within the genus *Formosa* and indicated that they represent a novel species.

## 2. Materials and Methods

### 2.1. Isolation and Maintenance of Bacterial Strains

Strains 4Alg 33^T^ and 3Alg 14/1 were isolated from the brown alga *Saccharina japonica* and the green alga *Ulva fenestrata*, respectively, collected from Troitsa Bay (42.622754, 131.121671), Gulf of Peter the Great, the Sea of Japan, Russia, using the dilution plating technique. For strain isolation, 0.1 mL of the seaweed homogenate was spread onto marine agar 2216 (MA; BD Difco^TM^, Sparks, MD, USA) plates. Following primary isolation and subsequent purification, the obtained strains were cultivated on the same medium at 28 °C and preserved at −70 °C in marine broth (MB; BD Difco^TM^, Sparks, MD, USA) supplemented with 20% (*v*/*v*) glycerol. Strains 4Alg 33^T^ and 3Alg 14/1 were deposited in the Collection of Marine Microorganisms (KMM), Russia, under numbers of KMM 3963^T^ and KMM 6136, respectively. The type strains *Formosa algae* KMM 3553^T^ and *F. agariphila* KMM 3901^T^ were provided by the KMM, whereas *Formosa maritima* KCTC 72531^T^, *F. undariae* KCTC 32328^T^ and *Xanthomarina spongicola* KCTC 22662^T^ were obtained from the Korean Collection for Type Cultures (KCTC), Republic of Korea. These strains were used as the reference strains in the parallel assays during this study. In addition, data from other type strains belonging to the neighboring genera on the genomic tree were included in tables as reference strains.

### 2.2. Phenotypic Characterization

The physiological, morphological, and biochemical characteristics of strains 4Alg 33^T^ and 3Alg 14/1 were investigated using established standard methods. The novel isolates were further analyzed with API 20NE, API 20E, API 50CH, and API ZYM test kits (bioMérieux, Marcy-l’Étoile, France) following the manufacturer’s protocols. All assays were carried out at 28 °C. Cell morphology was observed using a Zeiss Axio Scope.A1 light microscope (Zeiss, Jena, Germany) after 48 h of growth on MA at 28 °C. Gram-staining was performed as recommended by Gerhardt et al. [11]. Gliding motility was investigated as described by Bowman [12]. Oxidative or fermentative utilization of glucose was determined on Hugh and Leifson’s medium modified for marine bacteria [13]. Catalase activity was assessed by adding a 3% (*v*/*v*) H_2_O_2_ solution to bacterial colonies and observing for gas formation. Oxidase activity was determined using N,N,N,N-tetramethyl-p-phenylenediamine. The hydrolysis of agar, starch, casein, gelatin, chitin, DNA, L-tyrosine, urea, and Tweens 20, 40, and 80, as well as acid production from carbohydrates, nitrate reduction, and hydrogen sulfide formation, were examined following standard procedures [14]. The temperature range for growth was evaluated on MA between 4 °C and 42 °C at 1 °C intervals. Tolerance to NaCl was tested in medium containing (per liter of distilled water) 5 g of Bacto Peptone (BD Difco^TM^), 2 g of Bacto yeast extract (BD Difco^TM^), 1 g of glucose, 0.02 g of KH_2_PO_4_, and 0.05 g of MgSO_4_·7H_2_O supplemented with 0, 0.5, 1.0, 1.5, 2.0, 2.5, 3, 4, 5, 6, 8, 10, and 12% (*w*/*v*) NaCl. Growth within the pH range 5.0–11.0 was determined at 0.5 pH unit intervals. The presence of flexirubin-type pigments was examined as described previously [15]. Antibiotic susceptibility was determined using the standard disc diffusion plate method. Unless otherwise stated, discs contained the following antibiotics (µg per a disc): ampicillin (10), benzylpenicillin (10U), carbenicillin (100), cefalexin (30), cefazolin (30), chloramphenicol (30), erythromycin (15), doxycycline (10), gentamicin (10), kanamycin (30), lincomycin (15), nalidixic acid (30), neomycin (30), ofloxacin (5), oleandomycin (15), oxacillin (10), polymyxin B (300 U), rifampicin (5), streptomycin (30), tetracycline (5), and vancomycin (30).

### 2.3. Chemotaxonomic Characterization

Fatty acid methyl esters and polar lipids of strains 4Alg 33^T^ and 3Alg 14/1, together with their closest phylogenetic relatives, *F. undariae* KCTC 32328^T^, *F. maritima* KCTC 72531^T^, and *X. spongicola* KCTC 22662^T^, were extracted and analyzed as previously described using cells cultivated on MA at 28 °C [2]. Isoprenoid quinones were extracted with chloroform/methanol (2:1, *v*/*v*) and purified by TLC, employing n-hexane/diethyl ether (85:15, *v*/*v*) as the solvent system. Isoprenoid quinone composition was determined by HPLC on a Shimadzu LC-10A system (Shimadzu, Kyoto, Japan) equipped with a reversed-phase Supelcosil LC-18 column (15 cm × 4.6 mm; Supelco, Bellefonte, PA, USA). Acetonitrile/2-propanol (65:35, *v*/*v*) served as the mobile phase with a flow rate of 0.5 mL min^−1^ as described previously [16], and the column temperature was maintained at 40 °C. Quinones were detected by monitoring absorbance at 270 nm.

### 2.4. 16S rRNA Gene Sequence and Phylogenetic Analysis

Genomic DNA from strains 4Alg 33^T^ and 3Alg 14/1 was isolated using a NucleoSpin Tissue kit (Macherey–Nagel, Düren, Germany). The extracted DNA was subsequently used for PCR amplification of the 16S rRNA gene following a previously described protocol [17]. The obtained PCR fragments were sequenced and compared with the 16S rRNA gene sequences of validly published type strains using the EzBioCloud server, accessed on 13 October 2024 [18]. The phylogenetic relationships between the novel isolates and closely related type strains were analyzed through the GGDC web server (http://ggdc.dsmz.de/, accessed on 7 August 2025) [19] employing the DSMZ phylogenomics pipeline [20]. Maximum likelihood (ML) and maximum parsimony (MP) trees were constructed from aligned sequences using RAxML [21] and TNT [22], respectively, with 1000 bootstrap replications to assess branch robustness.

### 2.5. Whole-Genome Sequencing and Genome-Based Phylogenetic Analysis

The DNA libraries of strains 4Alg 33^T^ and 3Alg 14/1 were prepared using a Nextera DNA Flex kit (Illumina, San Diego, CA, USA) and sequenced on an Illumina MiSeq platform. For nanopore sequencing, the library was constructed with an SQK-NBD114.96 kit (Oxford Nanopore Technologies, Oxford, UK) and run on a MinION device equipped with a FLO-MIN 114 flow cell (Oxford Nanopore Technologies, Oxford, UK). Base calling was conducted using Dorado (v. 1.0.2). The obtained short and long reads were quality-filtered and trimmed with Trimmomatic (v. 0.39, quality > 30, length > 100) [23] and chopper (v. 0.10.0, quality > 16, length > 2000 for 3Alg14/1 and quality > 10 for 4Alg33) [24], respectively. The quality of processed reads was assessed with FastQC v. 0.11.8 (https://www.bioinformatics.babraham.ac.uk/projects/fastqc/, accessed on 15 July 2024) and NanoPlot v. 1.42.2 [25]. The hybrid assembly of the genomic sequences was performed using Unicycler v0.4.8 [26] with default parameters. The genome completeness and contamination were evaluated using CheckM v. 1.1.3 [27]. Sequencing depth was estimated utilizing SAMtools v. 1.3 [28]. Gene annotation was performed with RAST [29], PGAP [30], and Prokka [31].

Genome-based phylogenetic relationships were inferred with PhyloPhlAn v. 3.0.1 [32] employing the default set of 400 conserved proteins. An ML phylogenetic tree was reconstructed using RAxML v. 8.2.12 [21] under the LG + Γ substitution model, with 100 non-parametric bootstrap replicates. Pairwise average Nucleotide Acid Identity (ANI), Amino Acid Identity (AAI), and digital DNA–DNA hybridization (dDDH) values were calculated using FastANI [33], EzAAI [34], and TYGS platforms [35], respectively.

### 2.6. Functional Genomic Analysis

Pan-genome analysis of *Formosa* type strains, including metabolic profiling, was performed using the Anvi’o workflow v. 8 following the protocol described at https://merenlab.org/2016/11/08/pangenomics-v2/, accessed on 18 November 2024 [36]. Carbohydrate-active enzymes (CAZymes) and CAZyme-associated gene clusters were identified and annotated automatically using the dbCAN3 v.10 web server (http://dbcan-hcc.unl.edu/, accessed on 21 November 2025) [37,38]. Only CAZymes detected by at least two of the three search algorithms in dbCAN3 (HMMER, DIAMOND, and eCAMI) were retained for subsequent analysis. Biosynthetic gene clusters associated with secondary metabolite production were identified and annotated with antiSMASH v.8 with relaxed detection strictness (https://antismash.secondarymetabolites.org, accessed on 10 December 2025) [39]. Components of secretion systems were detected using MacSyFinder v.2.1.4 (TXSScan-1.1.3) [40]. Heatmaps and bar plots were generated using the R packages pheatmap v.1.0.12 and ggplot2 v.3.5.1 in RStudio v.2024.09.1+394 with R v.4.4.2. Figure fonts and layout were manually adjusted in Adobe Photoshop CC 2018 to improve clarity. Functional and ecological analyses were conducted using the Protologger web tool (https://www.protologger.de/, accessed on 1 December 2025) [41].

## 3. Results and Discussion

### 3.1. Phylogenetic Analyses

Analysis of the 16S rRNA gene sequences showed that the two new strains 4Alg 33^T^ (1159 bp long) and 3Alg 14/1 (1389 bp long) have 99.6% identity to each other and 98.9–99.1% identity to *F. undariae* WS-MY3^T^. The sequence identity values between 4Alg 33^T^ and other relatives of the genus *Formosa* ranged from 96.6% with *F. maritima* 1494^T^ to 99.0% with *Formosa arctica* IMCC 9485^T^, as well as 96.7% with *Bizionia arctica* SM1203^T^. The 16S rRNA gene sequences of strains 4Alg 33^T^ and 3Alg 14/1 were submitted to the GenBank under PQ573828 and PQ573827, respectively. They were 99.9–100% identical to those of four copies of the 16S rRNA gene retrieved from genome sequences of the strains.

**Figure 1:**
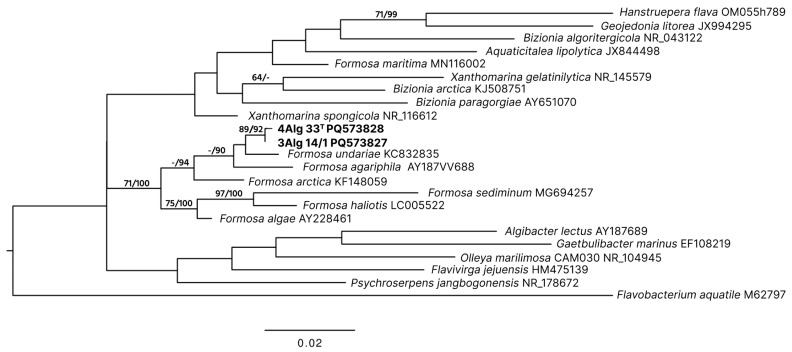
ML/MP 16S rRNA phylogenetic tree showing the positions of the novel strains 4Alg 33^T^ and 3Alg 14/1 (in bold) among type strains of the genus *Formosa* and closely related type strains of the family *Flavobacteriaceae*. The ML tree was constructed using the GTR + GAMMA substitution model. Bootstrap values (ML/MP) greater than 60%, based on 1000 replicates, are shown at branch nodes. The scale bar represents 0.02 substitutions per nucleotide position. GenBank accession numbers are provided alongside strain names.

Phylogenetic analysis based on 16S rRNA gene sequences showed that strains 4Alg 33^T^ and 3Alg 14/1 cluster together, representing a distinct lineage within the genus *Formosa* (Figure 1). Among the type strains of the genus *Formosa*, only the type strain of *F. maritima* lies outside the *Formosa* clade.

To date, the genus *Formosa* comprises seven species with validly published and correct names (https://lpsn.dsmz.de/genus/formosa, accessed on 15 December 2025), although genomic sequences are currently available for only six type strains, excluding *F. arctica*. Here, genomic sequences of type strains belonging to the genera *Formosa* and *Xanthomarina*, as well as [*Bizionia*] *arctica*, were subjected to a phylogenomic analysis. The genomic characteristics of *Formosa* species are presented in Table 1, whereas those of *Xanthomarina* type strains together with reclassified strains are listed in Table S1.

**Figure 2:**
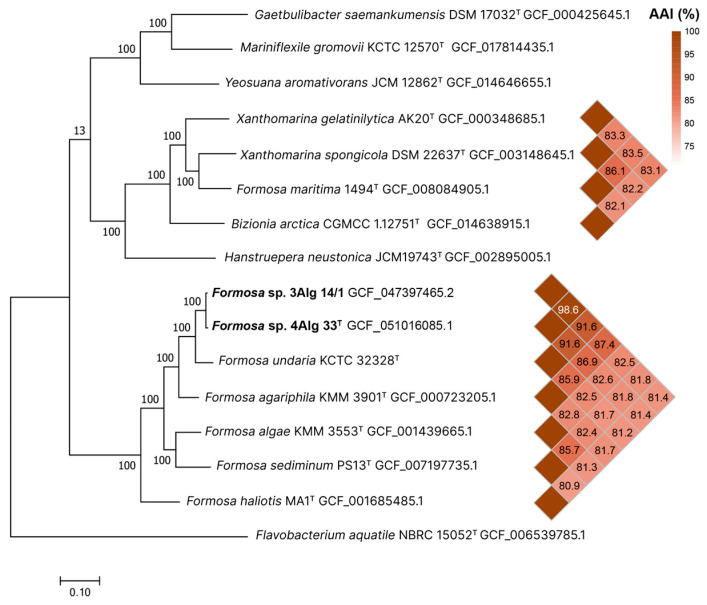
Maximum likelihood phylogenomic tree reconstructed from the concatenated sequences of 400 conserved proteins, depicting the phylogenetic placement of strains 4Alg 33^T^ and 3Alg 14/1 among the type strains of the genera *Formosa* and *Xanthomarina*, together with type species from other phylogenetically related genera. The bootstrap values of 100 replicates were employed. Scale bar represents 0.1 substitution per amino acid position. Strain *Flavobacterium aquatile* NBRC 15052^T^ was used as an outgroup.

A phylogenomic tree was reconstructed by the PhyloPhlAn method [32] from the concatenated sequences of 400 conserved proteins derived from the genomes of *Formosa* type strains and closely related genera (Figure 2). In this tree, the new strains 4Alg 33^T^ and 3Alg 14/1 occupy a separate taxonomic position, forming a common clade with the type strains *F. agariphila* KMM 3901^T^ and *F. undariae* KCTC 32328^T^. The ANI/AAI values between strains 4Alg 33^T^ and 3Alg 14/1 were 97.9%/98.6%, while the dDDH value was 85.3% (formula d4). The ANI/AAI values between 4Alg 33^T^ and type strains of the genus *Formosa* ranged from 80.8%/80.1% (*F. haliotis* MA1^T^) to 88.2%/91.4% (*F. undariae* KCTC 32328^T^), and the dDDH values were less than 40%. Despite the very high sequence similarity (99.1%) between the 16S rRNA gene sequences of strains 4Alg 33^T^ and *F. undariae* KCTC 32328^T^, ANI and dDDH values were lower than the generally accepted species delineation thresholds of 95–96% for ANI and 70% for dDDH. Therefore, in this case, genomic criteria are more decisive for species delimitation than criteria based on the 16S rRNA gene.

It is noteworthy that both type strains *F. maritima* 1494^T^ and *B. arctica* CGMCC 1.12751^T^ fall into the clade *Xanthomarina* with 100% branch support (Figure 2). Each strain forms its own distinct species-level lineage. The type strain *F. maritima* 1494^T^ shared AAI values of 86.1% with *X. spongicola* DSM 22637^T^, 83.4% with *X. gelatinilytica* AK20^T^ and less than 71.5% with *Formosa* type strains. The type strain *B. arctica* CGMCC 1.12751^T^ shared AAI values of 83.1% with *X. gelatinilytica* AK20^T^, 82.2% with *X. spongicola* DSM 22637^T^ and less than 72.8% with *Bizionia* type strains. The AAI value between *F. maritima* 1494^T^ and *B. arctica* CGMCC 1.12751^T^ was 82.1%. Therefore, both species should be transferred to the genus *Xanthomarina.*

Thus, the analysis combining the phylogenetic tree results and genome-relatedness indices clearly supported new strains 4Alg 33^T^ and 3Alg 14/1 as a novel species of the genus *Formosa* in the family *Flavobacteriaceae*.

### 3.2. Genomic Characteristics and Pan-Genome Analysis

**Figure 3:**
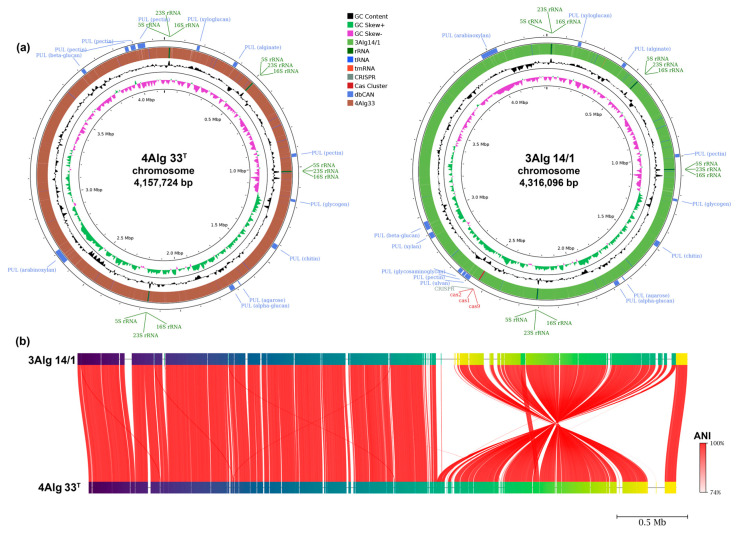
(**a**) Chromosomal maps of strains 4Alg33^T^ and 3Alg14/1 built using the Proksee server [43]. The inner scale indicates genome size in megabases (Mbp). From the innermost rings outward, the first two circles display a GC content (black) and GC skew (G−C)/(G+C) (in violet and green, respectively). The next circle shows CDSs strand. Moving outward, the blue circle shows PULs with the substrates indicated in brackets, annotated by the dbCAN3 server [37]. The figure also shows *rrn* operons and CRISPR-Cas system. (**b**) ANI visualization by FastANI. Each red line segment represents a reciprocal alignment between the two genomes, highlighting evolutionary conserved regions.

The complete circular genomes of 4Alg 33^T^ (=KMM 3963^T^) and 3Alg 14/1 (=KMM 6136) were assembled *de novo* for each strain (Figure 3). The obtained genomic characteristics aligned with the revised recommended minimal standards for the application of genomic data in prokaryotic taxonomy [42]. The genomes of 4Alg 33^T^ and 3Alg 14/1 are 4,157,724 bp and 4,316,096 bp in size, encoding 3570 and 3686 proteins, respectively, and with an overall G+C content of 34.5 mol% (Table 1).

The Proksee server was used to construct and visualize the chromosome maps of 4Alg 33^T^ and 3Alg 14/1 [43] (Figure 3a). To carry out genome annotations, the RAST [29] and Prokka [31] tool kits were utilized. Due to the chromosomal-level completeness of the genome assemblies, the number of *rrn* operons in each strain could be determined precisely, with all four operons located on the leading strands (Table 1, Figure 3a). It is noteworthy that the CRISPR-Cas system was detected in the 3Alg14/1 strain, while this system was absent in the type strain 4Alg33^T^. The ANI between the new strains amounted to 97.98%.

**Figure 4:**
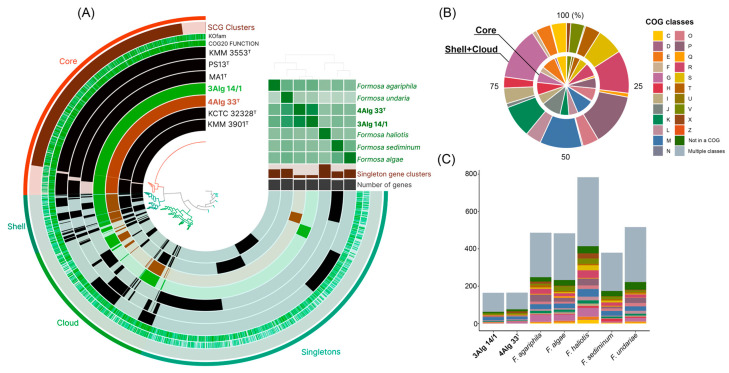
Pan-genome and functional COG classification in *Formosa*. (**A**) The pan-genome of *Formosa* genus members created using the anvi’o platform [36]. Functional classes of COG database predicted in the core and shell-cloud genomes (**B**) and distribution of unique genes into classes among *Formosa* strains (**C**). Circlular bars indicate the presence or absence of pan-genomic clusters across individual genomes. The heatmap in the upper right displays pairwise ANI and AAI values. The strain 4Alg 33^T^ is shown in brown, the strain 3Alg 14/1 in green, and other *Formosa* species in black. Additional data integrated into the figure include COG20 functional categories and KOfam module annotations. Description of COG one-letter codes can be found at https://www.ncbi.nlm.nih.gov/research/cog/cogcategory/, accessed on 23 January 2026.

To determine genus-related features, a pan-genome analysis of Formosa species (Table 1) was performed using orthologous clustering and metabolic pathway reconstruction with the anviὸ platform [36]. The Formosa pan-genome (Figure 4A) was composed of 7052 gene clusters (Euclidean distance; Ward’s linkage) with 25,680 gene calls. A total of 2386 core gene clusters were included in the core genome, covering 17,017 genes, among which 1917 were identified as single-copy genes (SCGs) with 13,419 gene calls. Composing the accessory shell and cloud, there were 513 (2553 genes) and 1255 (3135 genes) clusters, respectively. The singleton portion of the pan-genome included 2898 gene clusters (2975 genes). The largest number of singletons was observed in the genome of F. haliotis MA1T (755 gene clusters), while the smallest number was found in 4Alg 33T (157 clusters). All predicted gene clusters were annotated using COGs database (Figure 4B,C). Among the core cluster, the most represented functional classes were translation (J, 10.8%) and cell wall/membrane/envelope biogenesis (M, 9.5%). The most abundant COG classes in the accessory genome were carbohydrate metabolism and transport (G, 13.6%), inorganic ion transport and metabolism (P, 13.5%), general functional prediction only (R, 11%), cell wall/membrane/envelope biogenesis (M, 9.5%), and transcription (K, 8.3%) (Figure 4B). Most of the unique genes did not belong to any COG category. However, genes of categories G, M, P, V, and R prevailed (Figure 4C).

The observed gene patterns suggest that core functions support conserved cellular processes within the genus Formosa, while the accessory genome, in particular due to the enrichment of enzymes involved in carbohydrate metabolism and various transport proteins, reflects flexible adaptation to heterogeneous marine niches such as different algal hosts or particle-associated microenvironments. The prevalence of unassigned singletons further implies ongoing specialization toward lineage-specific substrates or interactions that remain to be elucidated.

Genes involved in central carbohydrate metabolism, including the Embden–Meyerhof pathway, gluconeogenesis, PRPP biosynthesis, pentose phosphate pathway, tricarboxylic acid cycle, pyruvate oxidation, and electron transport chain, were identified as complete metabolic pathways in all genomes, whereas the Entner–Doudoroff pathway was only partially represented. Protologger-based functional genome analysis [41] identified 3510 and 3580 coding sequences in strains 4Alg 33^T^ and 3Alg 14/1, respectively, including 108 transporter genes, 17 and 18 genes associated with secretion systems, and 726 and 724 unique enzyme genes. The genomes of both strains contain metabolic pathways associated with environmental adaptation, including folate (vitamin B9) biosynthesis from 7,8-dihydrofolate (EC 1.5.1.3), as well as pathways for the production of acetate (EC 2.3.1.8, 2.7.2.1), propionate (EC 2.3.1.8, 2.7.2.1), and L-glutamate (EC:6.3.1.2, 1.4.1.-). The predicted ability to synthesize zeaxanthin likely accounts for the yellow pigmentation observed in bacterial cells. In addition, the presence of a cbb_3_-type cytochrome c oxidase, inferred from the detection of subunits I/II (ccoNO), III, and IV, suggests adaptation to microaerobic conditions typical of benthic and host-associated marine environments.

### 3.3. In Silico Analysis of Hydrolytic and Biosynthetic Potentials

Coastal ecosystems with macroalgae and seagrasses are recognized as “blue carbon ecosystems” because of their capacity to capture and store substantial amounts of organic carbon [44]. Complex polysaccharides such as laminarin, alginate, ulvan, and others are primarily present in the cell walls of seaweeds, which can be degraded by marine bacteria. These bacteria synthesize polysaccharide-degrading enzymes that facilitate the depolymerization of algal polysaccharides. The resulting oligosaccharides are subsequently broken down into various intermediates, which are then used for energy conversion pathways [45]. Since both new strains, 4Alg 33^T^ and 3Alg 14/1, were isolated from different macroalgae, it is of interest to predict their hydrolytic potential and determine whether their genome reflects adaptation to the algal source.

**Figure 5:**
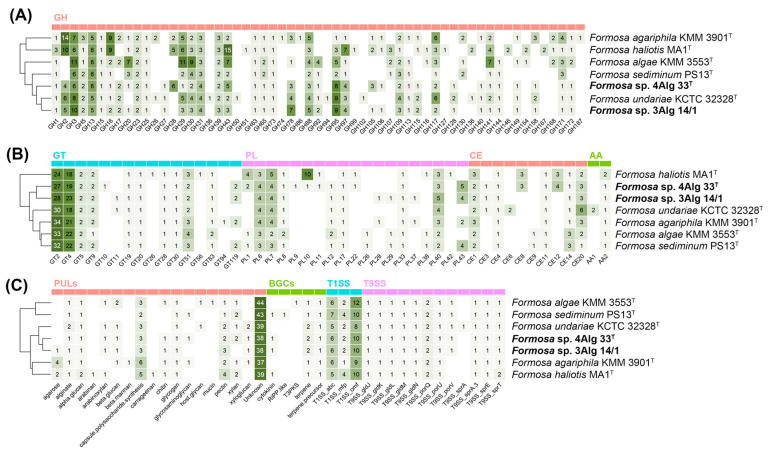
Heatmaps illustrating distribution of CAZymes (**A**,**B**), PULs, BGCs, and TSS (**C**) in 4Alg 33^T^, 3Alg 14/1, and the *Formosa* type strains. GH, glycoside hydrolase; GT, glycosyltransferase; CE, carbohydrate esterase; PL, polysaccharide lyase; AA, auxiliary activity; PUL, polysaccharide-utilizing locus; BGC, biosynthetic gene cluster; TSS, type secretion system.

Based on annotation using the dbCAN3 server [37], members of the genus *Formosa* encode a diverse repertoire of carbohydrate-active enzymes (CAZymes) and polysaccharide utilization loci (PULs), many of which were predicted to participate in the degradation of macroalgal polysaccharides. The proportion of genes encoding CAZymes in the analyzed genomes ranged from 4.55% to 5.92%, with the highest value predicted for *F. haliotis* MA1^T^. In the genomes of the novel strains 4Alg 33^T^ and 3Alg 14/1, CAZyme-related genes accounted for approximately 5.35% of the predicted coding sequences and were predominantly represented by glycoside hydrolases, followed by glycosyltransferases and polysaccharide lyases (Figure 5).

*Formosa* strains were enriched in glycoside hydrolase (GH) families associated with the degradation of diverse polysaccharides. GH29 and GH95, commonly linked to the hydrolysis of fucose-containing polysaccharides, were consistently represented across all analyzed genomes. Similarly, GH3, GH13, GH31, and GH97, associated with the degradation of α-glucans and related oligosaccharides, were prevalent. GH30 and GH43, which are associated with the degradation of xylan and related hemicellulosic polysaccharides, were widely detected in the novel strains. In addition, strains 4Alg 33^T^, 3Alg 14/1, and KCTC 32328^T^ uniquely encoded GH27, which includes α-galactosidases involved in the degradation of galactose-containing algal polysaccharides. Interesting to note, GH28, commonly associated with polygalacturonase activity and pectin-like substrate degradation, was detected exclusively in strain 4Alg 33^T^.

Glycosyltransferases (GTs), particularly families GT2, GT4, and GT51, were consistently detected in all strains, with GT2 and GT4 occurring in high copy numbers (up to 34 per genome), suggesting an important role in the biosynthesis of exopolysaccharides, cell wall polymers, and glycoproteins.

The majority of annotated polysaccharide lyases (PLs) were assigned to families PL6, PL7, and PL40, which are commonly associated with the depolymerization of alginate and ulvan, the predominant structural polysaccharides of brown and green algae, respectively. Among carbohydrate esterases (CEs), family CE20 was most frequently detected and is linked to the deacetylation of algal xylans, a modification that may increase polysaccharide accessibility in marine environments. Furthermore, the genomes of strains 4Alg 33^T^ and MA1^T^ encoded CAZymes belonging to families GH28, PL10, CE8, and CE12 that are predicted to be involved in pectin degradation. The presence of these enzymes suggests a potential capacity to utilize plant-derived polymeric substrates in coastal habitats influenced by terrestrial inputs. Approximately half of the annotated GH, PL, and CE proteins predicted by dbCAN3 contained a signal peptide, suggesting their targeting to the periplasmic or extracellular space and supporting a role in the extracellular processing and turnover of complex polysaccharides in marine environments.

A large number of PULs were detected within the *Formosa* genus, comprising genes related to the modification and degradation of various marine polysaccharides; however, the majority of these loci could not be functionally annotated, indicating substantial unexplored metabolic potential.

It is known that laminarin is widely distributed in brown algae but is often found in diatoms and golden algae [46]. The macroalgal polysaccharide laminarin primarily consists of a linear backbone of 20–30 β-1,3-linked D-glucopyranose residues, with branching chains formed by β-1,6-linked D-glucopyranose units. Using the dbCAN server, PULs capable of degrading beta-glucan were identified in both strains 4Alg 33^T^ and 3Alg 14/1. A detailed analysis of these loci revealed genes encoding GH16, GH17, and GH30, responsible for laminarin utilization [47]. Another polysaccharide-utilizing cluster, found in both strains 4Alg 33^T^ and 3Alg 14/1, was predicted to be an alginate-degrading cluster. Alginate is a linear, anionic polysaccharide from brown algae, made of repeating units of two uronic acids: β-D-mannuronic acid and α-L-guluronic acid linked by (1→4) bonds. These PUL genes, encoding PL-6, -7, and -17, are well-characterized alginate lyases [48,49,50].

**Figure 6:**
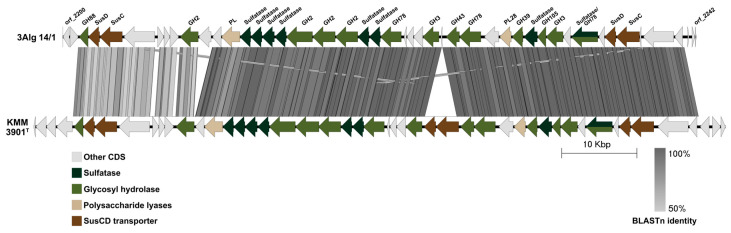
Genomic organization and genetic relatedness of ulvan-utilizing loci between 3Alg 14/1 and *F. agariphila* KMM 3901^T^ strains. BLAST version 2.16.0+ identity is represented by grayscale shading ranging from 100% to 50%.

Ulvan-utilizing PUL was detected in 3Alg 14/1 and absent from 4Alg 33^T^. This difference is not surprising, as strain 3Alg 14/1 was isolated from the green alga *Ulva fenestrata*. Ulvan is a branched sulfated polysaccharide consisting of repeating disaccharide units, where D-glucuronic acid or L-iduronic acid is linked to L-rhamnnose 3-sulfate through β-1,4 and α-1,4-bonds, respectively, the last residues form an α-1,4-linked backbone, giving the polymer its characteristic branched configuration. GH105 and PL28 enzymes were shown to be key enzymes for the degradation of ulvan [51]. The ulvan-degrading cluster of strain 3Alg 14/1 was compared with the same cluster previously described in *F. agariphila* KMM 3901^T^ isolated from the green alga *Acrosiphonia sonderi* (Figure 6). Both clusters have a similar structure, but the KMM 3901^T^ strain contains genes encoding the additional SusCD transporter. The 4Alg 33^T^ genome also contained three enzymes belonging to the GH105 family, but these were predicted to be related to pectin utilization. Furthermore, PL28 was absent from the 4Alg 33^T^ genome, indicating that this strain is unable to utilize ulvan.

Thus, despite the high sequence similarity between strains 4Alg 33^T^ and 3Alg 14/1, their genomes differ at several PULs, determined by different substrate preferences in their ecological niche, namely, green or brown algae. It also indicates the ecological importance of the new *Formosa* species in the global carbon cycle in marine ecosystems.

Analysis using TXSScan [40] predicted the presence of two types of secretion systems (TSSs) in all studied *Formosa* genomes (Figure 5C). An almost complete gene set encoding the type IX secretion system (T9SS), a hallmark of members of the phylum *Bacteroidota*, was identified. The mandatory gene *sprA* was not detected due to low sequence similarity to known homologues. The T9SS is essential for the secretion of cell-surface and extracellular proteins, including some CAZymes, and is also required for gliding motility. In agreement with these genomic predictions, gliding motility was observed in *Formosa* strains during phenotypic characterization. In addition to T9SS, T1SS was detected in all analyzed genomes. T1SS is commonly found in Gram-negative bacteria and facilitates the direct, one-step transport of substrates from the cytoplasm to the extracellular environment. The genomes of strains 4Alg 33^T^ and 3Alg 14/1 encoded up to ten copies of the core T1SS components, comprising the ATP-binding cassette transporter, membrane fusion protein, and outer membrane factor.

The biosynthetic gene cluster (BGC) repertoire of the genus *Formosa* was analyzed using the antiSMASH server [39]. Two types of BGCs were detected in all examined genomes and were predicted to be involved in the biosynthesis of terpenes and terpene precursors (Figure 5C). In the genomes of the novel strains, as well as strains MA1^T^ and PS13^T^, an additional gene cluster (cytokinin) was identified. This cluster may play a role in microbe–host interactions or environmental signaling. No gene clusters associated with flexirubin-type pigment biosynthesis, which are common among *Bacteroidota* members, were detected in all analyzed genomes. This finding is supported by phenotypic characteristics of *Formosa* strains (Table 2). However, gene clusters putatively related to carotenoid biosynthesis were predicted in all genomes, suggesting that carotenoids may contribute to the yellow-to-orange pigmentation observed in the strains.

Ecological distribution analysis using the Protologger web tool did not identify any metagenome-assembled genomes (MAGs) corresponding to novel strains, indicating limited representation in current genomic databases [41]. However, 16S rRNA gene-based screening revealed 4Alg 33^T^- and 3Alg 14/1-like sequences in coral-associated metagenomes (29.6% and 27.0%, respectively), followed by marine sediment (27.2% and 21.2%) and marine (19.3% and 16.7%) datasets.

The observed distribution agrees with the isolation sources of the strains and with the known ecology of the genus *Formosa*, whose representatives are largely marine and commonly associated with coastal and host-related environments.

### 3.4. Phenotypic Characterization of New Strains

Strains 4Alg 33^T^ and 3Alg 14/1 were Gram-stain-negative, aerobic, rod-shaped, yellow-pigmented and motile by gliding. Both strains were positive for oxidase activity, hydrolysis of aesculin, agar and gelatin, and acid production from D-fructose, D-galactose, D-glucose, maltose, D-mannose, D-xylose and *N*-acetyl-glucosamine (Table 2). The new isolates shared some common features with their nearest neighbors, which supported affiliation of them with the genus *Formosa* (Table 2). However, unlike their closest relative species, *F. undariae* 32328^T^, strains 4Alg 33^T^ and 3Alg 14/1 possessed a gliding motility, agar and gelatin hydrolysis as well as esterase (C4), trypsin, α-chymotrypsin, N-acetyl-*β*-glucosamine and α-fucosidase activities. A type of metabolism, the ability to degrade agar, to form acid from D-galactose and L-rhamnose, and to produce cysteine arylamidase, trypsin, α-chymotrypsin and α-fucosidase helped to differ the novel strains from *F. algae* KMM 3553^T^. A set of phenotypic properties, including the presence of acid formation from L-rhamnose and α-glucosidase as well as the incapability of the both strains to grow under facultatively anaerobic conditions and to utilize L-arabinose and D-lactose clearly separated them from *F. agariphila* KMM 3901^T^ (Table 2). The detailed phenotypic characteristics of strains 4Alg 33^T^ and 3Alg 14/1 are summarized in Table 2 and described in the species description section.

The cellular fatty acid profiles of strains 4Alg 33^T^ and 3Alg 14/1 contained iso-C_15:0_, iso-C_17:0_ 3-OH, iso-C_16:0_ 3-OH, C_16:1_ ω7c, iso-C_15:1_, C_16:0_, C_15:0_ and C_15:1_ ω6c as the predominant fatty acids (>5%) and were similar to those of the reference strains (Table 3). Along with this, distinctiveness in the relative abundances of C_16:0_ and iso-C_15:0_ 3-OH fatty acids were observed between the novel strains and their closest phylogenetic relatives, *F. algae* KMM 3553^T^ and *F. agariphila* KMM 3901^T^. Also noteworthy is the presence of fatty acids C_15:0_ 3-OH and C_17:0_ 3-OH in the above-mentioned type strains in comparison with strains 4Alg 33^T^ and 3Alg 14/1 (Table 3).

### 3.5. Reclassification of Formosa maritima and Bizionia arctica as New Xanthomarina Species

Despite the 16S rRNA gene sequence similarity between strain 4Alg 33^T^ and *X. spongicola* KCTC 22662^T^ being 97.7%, the phylogenomic tree revealed that the aforementioned strains formed different clusters (Figure 2). Moreover, the species *F. maritima* is closely related to species of the genus *Xanthomarina*, with ANI values ranging from 80.5 to 84.2% and AAI values of 82.1–83.1%. It forms a distinct evolutionary lineage within the genus *Xanthomarina* (Figure 2).

Indeed, consistently with the results of the phylogenetic analysis, phenotypic characteristics of *F. maritima* are similar to those of species of the genus *Xanthomarina*, including the respiratory type of metabolism, presence of gliding motility, oxidase activity, hydrolysis of gelatin and production of flexirubin-type pigments, as well as the absence of aesculin and agar hydrolysis (Table 4). However, *F. maritima* KCTC 72531^T^ could be distinguished from *Xanthomarina gelatinolytica* AK20^T^ by the minimal temperature for growth, arginine dihydrolase activity, urea hydrolysis, D-glucose oxidation and utilization and the DNA G+C content value. A set of such phenotypic features as nitrate reduction, hydrolysis of casein, starch, L-tyrosine and Tween 80, utilization of several sugars and acid phosphatase and α-galactosidase activities strongly supported the discrimination of *F. maritima* from *X. spongicola* (Table 3).

The fatty acid profile of strain *F. maritima* KCTC 72531^T^ was in accordance with that of *X. spongicola* KCTC 22662^T^ and *X. gelatinolytica* AK20^T^ (Table 4). Moreover, the polar lipid composition of strain *F. maritima* KCTC 72531^T^ was similar to that of *X. spongicola* and included PE, two unidentified aminolipids (AL 1-2) and four unidentified lipids (L1-4) (Figure S2). The above-mentioned data clarified a precise taxonomic position of the species *F. maritima* and strongly justified its inclusion to the genus *Xanthomarina* as *Xanthomarina maritima* comb. nov.

Recently, the phylogenomic analysis of the genus *Bizionia* revealed that the type strain *B. arctica* SM1203^T^ is the closest relative of *Xanthomarina* members, as well as *F. maritima* KCTC 72531^T^ [55] (Figure 2). The ANI values between *B. arctica* SM1203^T^ and *Xanthomarina* type strains were 79.8–80.9%, whereas the AAI values were 82.1–83.1%. *B. arctica* had many phenotypic properties in common with the validly published species of the genus *Xanthomarina*, but it could be differentiated from them by the absence of gliding motility, oxidase activity and production of flexirubin-type pigments (Table 4). However, using the antiSMASH server, a flexirubin biosynthesis gene cluster was discovered in the genome of *B. arctica* SM1203^T^, similar to the *Flavobacterium johnsoniae* UW101 gene cluster.

The fatty acid profile of *B. arctica* SM1203^T^ demonstrated some differences in the proportions of several fatty acids in comparison with those of the type strains of the genus *Xanthomarina*. However, all prevalent fatty acids were presented herein, including fatty acids iso-C_15:0_, iso-C_17:0_ 3-OH and iso-C_15:1_ (Table 5). Thus, the results of the phylogenomic and phenotypic analyses clarified the precise taxonomic position of species *B. arctica* and strongly supported transferring the species *B. arctica* to the genus *Xanthomarina* and its reclassification as *Xanthomarina arctica* comb. nov.

## 4. Conclusions

Brown algae *Saccharina japonica* and green algae *Ulva fenestrata* are common inhabitants of the Far East’s coastal waters in the Sea of Japan. During the study of the cultured microbial diversity of these macroalgae two novel strains, 4Alg 33^T^ and 3Alg 14/1 were identified. A comprehensive CAZyme analysis showed that strain-specific differences in PUL content can reflect the sources of their initial isolation. The strain 4Alg 33^T^, isolated from brown algae, carries multiple PULs for alginate and laminarin degradation, two hallmark polysaccharides of brown algal cell walls. The presence of PL6, PL7, and PL17, along with GH16 and GH17 (laminarinases), reflects its adaptation to the utilization of brown-algal detritus. In contrast, 3Alg 14/1, isolated from green algae, possesses an ulvan-degrading cluster with GH105 and PL28, which is absent from 4Alg 33^T^. This directly links its genomic traits to its ecological niche: the ability to utilize ulvan provides a selective advantage at or near *Ulva* surfaces, where such polysaccharides predominate. The presence of genes responsible for the breakdown of pectin and xylan in both genomes further indicates metabolic flexibility, possibly allowing these strains to process mixed organic matter in coastal habitats where macroalgae and terrestrial organisms converge.

Moreover, the phylogenetic analyses, taken together with chemotaxonomic data obtained in this study, suggest that strains 4Alg 33^T^ and 3Alg 14/1 belong to the genus *Formosa* and represent a novel species within the genus, for which the name *Formosa bonchosmolovskayae* sp. nov. is proposed.

In addition, the combination of the genomic and phenotypic characteristics strongly supports the affiliation of the species *Formosa maritima* and *Bizionia arctica* with the genus *Xanthomarina* and permits their reclassification as *Xanthomarina maritima* comb. nov. and *Xanthomarina arctica* comb. nov.

**Description of** ***Formosa bonchosmolovskayae*** **sp. nov.**

*Formosa bonchosmolovskayae* (bonch.os.mo.lovs’ka.yae. N.L. gen. fem. n. bonchosmolovskayae, of Bonch-Osmolovskaya, in honor of famous Russian microbiologist Elizaveta A. Bonch-Osmolovskaya, who has made a significant contribution to the investigation of environmental microbiology).

Cells are Gram-negative, strictly aerobic, motile by gliding and rod-shaped (approximately 0.4–0.8 μm in diameter and 1.2–5.2 μm in length). On marine agar, colonies are 2–3 mm in diameter, circular, with entire edges, shiny, yellow pigmented and slightly sinking into agar. Growth occurs at 4–34 °C (optimum, 25–28 °C), at pH 5.5–9.0 (optimum, pH 7.5) and with 0–8% NaCl (optimum, 1–3%). Arginine dihydrolase, lysine decarboxylase, ornithine decarboxylase and tryptophan deaminase activities are absent. Aesculin, agar and gelatin are hydrolyzed, but casein, chitin, L-tyrosine, Tween 80, DNA and urea are not degraded. Hydrolysis of starch and Tweens 20 and 40 are strain-dependent. Acid is produced from D-arabinose, D-fructose, D-galactose, D-glucose, D-mannose, rhamnose, sucrose, D-xylose, and mannitol but not from L-arabinose, raffinose, ribose, trehalose, inositol, sorbitol, D-cellobiose, lactose, maltose, melibiose and N-acetyl-glucosamine. Acid production from glycogen is strain-dependent. Utilization of gluconate is strain-dependent. Nitrate is not reduced to nitrite. Hydrogen sulfide and indole are not produced. Production of acetoin is strain-dependent. Flexirubin-type pigments are not formed. In API 20NE gallery, positive results are obtained for aesculin, gelatin and PNPG hydrolysis and utilization of maltose, mannose and mannitol as sole carbon sources. Utilization of gluconate is strain-dependent. In API 20E, positive results are obtained for ONPG and gelatin hydrolysis and acid formation from D-glucose. Production of acetoin and acid formation from mannitol, sucrose and melibiose are strain-dependent. In API 50CH, a positive result is obtained for acid formation from D-arabinose, D-xylose, galactose, glucose, fructose, mannose, rhamnose, aesculin, amygdalin and L-fucose and utilization of N-acetylglucosamine, maltose and glycogen. Acid formation from mannitol and N-acetylglucosamine is strain-dependent. In the API ZYM gallery, alkaline phosphatase, esterase (C4), esterase lipase (C8), leucine arylamidase, cystine arylamidase, valine arylamidase, trypsin, α-chymotrypsin, acid phosphatase, naphtol-AS-BI-phosphohydrolase, α-glucosidase, N-acetyl-β-glucosaminidase and α-fucosidase activities are present, but lipase (C14) and β-glucuronidase activities are absent. Activity of α-galactosidase, β-galactosidase, β-glucosidase and α-mannosidase are strain-dependent. The predominant fatty acids are iso-C_15:0_, iso-C_17:0_ 3-OH, iso-C_16:0_ 3-OH, C_16:1_ ω7c, iso-C_15:1_, C_16:0_, C_15:0_ and C_15:1_ ω6c. The polar lipid profile consists of phosphatidylethanolamine, one unidentified aminolipid and three unidentified lipids. The major respiratory quinone is MK-6. The DNA G+C content of the type strain is 34.5 mol%.

The type strain 4Alg 33^T^ (=KCTC 72008^T^=KMM 3963^T^) was isolated from the brown alga *Saccharina japonica* collected from Troitsa Bay, Gulf of Peter the Great, Sea of Japan (also is known as East Sea), Pacific Ocean, Russia (42.622754, 131.121671).

The DDBJ/GenBank accession number for the 16S rRNA gene sequence of strain 4Alg 33^T^ is PQ573828.

The GenBank accession number for the whole-genome sequence of strain 4Alg 33^T^ is CP174605.1.

**Description of** ***Xanthomarina maritima*** **comb. nov.**

*Xanthomarina maritima* (ma.ri′ ti. ma. L. fem. adj. *maritima* of or belonging to the sea, maritime, referring to coastal sediment from which the type strain was isolated).

Basonym: *Formosa maritima* Cao et al. 2020.

The description of this species is the same as that provided by Cao et al. (2020).

The type strain 1494^T^ (=KCTC 72531^T^=MCCC 1H00385^T^) was isolated from marine sediment collected off the coast of Weihai, PR China. The DNA G+C content of the type strain is 31.1 mol%. Genome size is 3.0 Mb.

**Description of** ***Xanthomarina arctica*** **comb. nov.**

*Xanthomarina arctica* (arc′ ti.ca. L. fem. adj. arctica northern, from the Arctic).

Basonym: *Bizionia arctica* Li et al. 2015.

The description of this species is the same as that provided by Li et al. (2015).

The type strain SM1203^T^ (=CGMCC 1.12751^T^=JCM 30333^T^) was isolated from surface seawater of Kongsfjorden, Svalbard. The genomic DNA G+C content of the type strain is 33.0 mol%. Genome size is 3.9 Mb.

## Figures and Tables

**Table 1:** Genomic features of novel strains 4Alg 33^T^ and 3Alg 14/1, and type strains of the genus *Formosa*.

Feature	1	2	3	4	5	6	7	8
Assembly level	Chromosome	Chromosome	Contig	Chromosome	Contig	Chromosome	Contig	Scaffold
Genome size (Mb)	4.2	4.3	4.4	4.2	4.3	3.9	4.3	3
Number of contigs	1	1	4	9	82	1	1	76
G+C Content (mol%)	34.5	34.5	34.1	33.5	33.5	32	34.5	31
N50 (Mb)	4157.7	4316.1	4407.2	2500	108.4	3900	4300	141.1
L50	1	1	1	1	13	1	1	8
Coverage (x)	180	196	150	53	22	101	25	644
Total genes	3545	3663	3962	3623	3693	3445	3711	2838
Protein-coding genes	3459	3579	3897	3541	3613	3367	3542	2775
rRNAs (5S/16S/23S)	4/4/4	4/4/4	5/5/5	4/3/3	1/1/1	4/4/4	4/4/4	1/1/3
tRNA	47	47	50	45	41	46	46	36
checkM completeness (%)	100.0	99.35	99.01	99.35	99.68	99.84	95.07	99.35
checkM contamination (%)	0.11	1.29	1.83	0	1.08	0.82	0.49	0.16
WGS project	-	JBJJID02	-	-	LMAK01	-	BDEL01	VSFC01
Genome assembly name	ASM5101608v1	ASM4739746v2	PRJNA1402693	FAGA1	ASM143966v1	ASM719773v1	ASM168548v1	ASM808490v1

Strains: **1**, 4Alg 33^T^; **2**, 3Alg 14/1; **3**, *F. undariae* KCTC 32328^T^; **4**, *F. agariphila* KMM 3901^T^; **5**, *F. algae* KMM 3553^T^; **6**, *F. sediminum* PS13^T^; **7**, *F. haliotis* MA1^T^; **8**, *F. maritima* 1494^T^.

**Table 2:** Differential characteristics of strains 4Alg 33^T^and 3Alg 14/1 compared to their closely related species of the genus *Formosa*.

Feature	1	2	3	4
Source of isolation	Brown and green algae	Brown alga	Brown alga	Green alga
Type of metabolism	A	A	F	F
Gliding motility	+	−	+	+
Temperature range for growth (°C):	4–34	4–30	4–34	4–33
Salinity range for growth (% NaCl):	0–8	0–9	0–8	1–8
Degradation of:				
Agar	+	−	−	+
Gelatin	+	−	+	+
Acid formation from:				
D-Cellobiose, D-lactose	−	+	−	−
D-Galactose	+	+	−	+
L-Rhamnose	+	+	−	−
Glycerol	−	ND	+	−
Utilization of:				
L-Arabinose, D-lactose	−	+	−	+
Enzyme activities (API ZYM):				
α-Glucosidase	+	+	+	−
Cysteine arylamidase,	+	+	−	+
Esterase (C4), N-acetyl-*β*-glucosaminidase	+	−	+	+
Trypsin, α-chymotrypsin, α-fucosidase	+	−	−	+
Susceptibility to:				
Ampicillin, carbenicillin	+	−	+	+
Benzylpenicillin	+	−	−	+
Oleandomycin	+	+	−	−
Cefazolin	+	−	+	+
DNA G+C content (mol%)	34.5	37.3	33.5	33.5
Oxidase	+	+	+	+
Catalase	+	+	+	−

Strains: 1, 4Alg 33^T^ and 3Alg 14/1; 2, *F. undariae* KCTC 32328^T^; 3, *F. algae* KMM 3553^T^; 4, *F. agariphila* KMM 3901^T^. The strains were positive for the following tests: catalase and oxidase activities; hydrolysis of aesculin; acid production from D-fructose, D-glucose, maltose, D-mannose, D-xylose and N-acetyl-glucosamine; utilization of D-fructose, D-galactose, D-glucose, maltose, D-mannose, D-xylose and D-mannitol; alkaline phosphatase, esterase lipase (C8), leucine arylamidase, valine arylamidase, acid phosphatase and naphthol-AS-BI-phosphohydrolase activities; susceptibility to cefalexin, chloramphenicol, doxycycline, erythromycin, lincomycin, ofloxacin, rifampicin, tetracycline and vancomycin, and resistance to gentamicin, kanamycin, neomycin, oxacillin, polymyxin and streptomycin. The strains were negative for the following tests: nitrate reduction; hydrolysis of casein, chitin, L-tyrosine, Tween 80, DNA and urea; H_2_S and indole production; flexirubin-type pigments production; sucrose and citrate utilization; lipase (C14) and *β*-glucuronidase activities. A, aerobic; F, facultatively anaerobic; +, positive; −, negative; ND, not determined.

**Table 3:** Fatty acid profile of strains 4Alg 33^T^ and 3Alg 14/1 and the type strains of the closely related members of the genus *Formosa*.

Fatty Acid	1	2	3	4	5
Saturated straight-chain:					
C_15:0_	5.9	7.6	6.3	18.5	13.0
C_16:0_	8.0	7.5	8.0	2.6	4.3
C_18:0_	3.9	2.5	4.3	Tr	1.2
Unsaturated straight-chain:					
C_15:1_ ω6*c*	4.9	5.5	6.3	13.5	7.6
C_16:1_ ω7*c*	7.8	11.5	8.5	5.8	6.1
Branched-chain:					
iso-C_14:0_	Tr	1.1	1.6	2.0	1.7
iso-C_15:0_	18.3	14.0	15.5	12.6	15.7
anteiso-C_15:0_	2.7	4.3	3.4	4.1	6.7
iso-C_15:1_	10.6	7.8	9.7	8.6	6.6
iso-C_16:0_	4.0	4.3	2.3	2.3	3.0
iso-C_16:1_	3.6	2.3	1.9	2.1	2.0
iso-C_17:1_ ω8*c*	1.4	1.2	Tr	1.3	1.5
Hydroxy-substituted:					
iso-C_15:0_ 3-OH	2.2	2.7	2.8	9.2	10.3
iso-C_16:0_ 3-OH	8.6	10.8	5.4	9.0	10.6
iso-C_17:0_ 3-OH	15.4	14.5	21.4	6.4	8.3

Strains: 1, 4Alg 33^T^; 2, 3Alg 14/1; 3, *F*. *undariae* KCTC 32328^T^; 4, *F. algae* KMM 3553^T^ [2]; 5, *F. agariphila* KMM 3901^T^ [2]. Tr, trace (<1%); Summed feature 3 consists of iso-C15:0 2-OH and/or C161 ω7c that could not be separated by the microbial identification system. The polar lipid composition of strains 4Alg 33^T^ and 3Alg 14/1 included phosphatidylethanolamine (PE), one unidentified aminolipid (AL1) and three unidentified lipids (L1-3) (Figure S1) and was in line with that of the reference strains and other type strains of the validly published species of the genus *Formosa* (Figure S1) [7,8,9]. However, the presence of third unidentified lipid (L3) differed the novel strains from their nearest neighbor, *F. undariae* (Figure S1). The major respiratory quinone of the strains studied was MK-6.

**Table 4:** Differential characteristics of *Formosa maritima* KCTC 72531^T^, *Bizionia arctica* SM1203^T^ and *Xanthomarina* species.

Feature	1	2	3	4
Source of isolation	Marine sediment	Seawater	Marine sponge	Seawater
Temperature range for growth (°C):	4–37 *	4–30	15–35 **	10–40
Salinity range for growth (% NaCl):	0–8 *	1–6	1–5 **	0.5–7.5
Gliding motility	+	−	+	+
Oxidase	+	−	+	+
Arginine dihydrolase	−	+	−	+
Nitrate reduction	+	−	+	−
Flexirubin-type pigment production	+	−	+	+
Hydrolysis of:				
Casein	−	+	+	−
Starch	−	−	+	−
Tween 80	+	−	−	+
Tyrosine	−	ND	+	ND
Urea	−	−	−	+
Acid formation from D-glucose	+	+	+	−
Utilization of:				
L-Arabinose	− *	−	+ **	ND
D-Glucose	+ *	−	+ **	−
Maltose, D-mannose	− *	−	+ **	−
N-acetyl-glucosamine, gluconate	− *	−	+ **	ND
Enzyme activities (API ZYM):				
Acid phosphatase	+ *	+	− **	ND
α-Galactosidase	− *	−	+ **	−
Susceptibility to:				
Ampicillin	−	+	+	+
Carbenicillin, cefalexin	−	+	+	ND
Neomycin	−	−	+	−
Tetracycline, vancomycin	−	+	+	−
Streptomycin	+	−	−	ND
DNA G+C content (mol%) *	35.5	31	31	33

Strains: 1, *F. maritima* KCTC 72531^T^ (data from this study); 2, *B.* *arctica* SM1203^T^, data from [52]; 3, *X. spongicola* KCTC 22662^T^ (data from this study); 4, *X. gelatinolytica* AK20^T^, data from [53]. * Data from [6]; ** data from [54]. The strains were positive for the following tests: the respiratory type of metabolism; alkaline phosphatase, esterase lipase (C8), cysteine arylamidase and catalase activities; hydrolysis of gelatin; susceptibility to chloramphenicol, and resistance to gentamycin, kanamycin and polymyxin. The strains were negative for the following tests: hydrolysis of agar and aesculin; utilization of D-mannitol and citrate; *β*-glucosidase, *β*-galactosidase, *β*-glucuronidase, N-acetyl-*β*-glucosaminidase and α-fucosidase activities. All data were from this study unless otherwise indicated. +, positive; −, negative; ND, not determined.

**Table 5:** Fatty acid profiles of strain *Formosa maritima* KCTC 72531^T^, *Bizionia arctica* SM1203^T^ and *Xanthomarina* species.

Fatty Acid	1	2	3	4
Saturated straight-chain:				
C_15:0_	4.6	5.3		1.7
C_16:0_	5.6	Tr		6.1
C_18:0_	2.2			3.1
Unsaturated straight-chain:				
C_15:1_ ω6*c*	1.2	Tr		Tr
C_16:1_ ω7*c*	3.5		7.9	2.6
Branched-chain:				
iso-C_14:0_	2.7	3.4		1.3
iso-C_15:0_	25.0	25.5	39.3	25.6
anteiso-C_15:0_	4.7	14.2		3.8
iso-C_15:1_	15.3	20.3	29.5	8.6
anteiso-C_15:1_	-	3.4	-	-
iso-C_16:0_	4.6	Tr		1.7
iso-C_16:1_	2.9	1.2		Tr
iso-C_17:1_ ω8*c*	2.3			1.8
iso-C_17:1_ ω9*c*		Tr	3.8	
Hydroxy-substituted:				
C_15:0_ 2-OH	-	1.7	-	-
iso-C_15:0_ 3-OH	2.4	2.7	6.1	2.5
iso-C_16:0_ 3-OH	5.0	3.9		2.5
C_17:0_ 2-OH	-	1.2	-	-
iso-C_17:0_ 3-OH	16.2	4.8	13.4	35.4

Strains: 1, *Formosa maritima* KCTC 72531^T^ (this study): 2, *Bizionia arctica* SM1203^T^ (data from [51]); 3, *Xanthomarina gelatinolytica* AK20^T^ (data from [52]); 4, *Xanthomarina spongiae* KCTC 22662^T^ (this study). Tr, trace (<1%); - not detected.

## Data Availability

The original data presented in the study are openly available in DDBJ/GenBank at PQ573828, PQ573827, CP174605.1, CM137262.1.

## Supplementary Materials

The following supporting information can be downloaded at: https://www.mdpi.com/article/10.3390/microorganisms14020328/s1, Table S1: Genomic features of type strains of *Xanthomarina* genus, *F. maritima* 1494^T^ 4, and *B. arctica* CGMCC 1.12751^T^; Figure S1: Two-dimensional thin-layer chromatogram of polar lipids extracted from strain 4Alg 33^T^ (a), 3Alg 14/1 (b), *Formosa undariae* KCTC 32328^T^ (c), *Xanthomarina spongicola* KCTC 22662^T^ (d) and *Formosa maritima* KCTC 72531^T^ (e). PE, phosphatidylethanolamine; AL1-2, unidentified aminolipids, L1-4, unidentified lipids; Figure S2: Two-dimensional thin-layer chromatogram of polar lipids extracted from strain *Formosa maritima* KCTC 72531^T^ (a) and *Xanthomarina spongicola* KCTC 22662^T^ (b). PE, phosphatidylethanolamine; AL1-2, unidentified aminolipids, L1-4, unidentified lipids.
