# Supplementary material for: Genome Analysis and Characterization of Formosa bonchosmolovskayae sp. nov. Isolated from Brown and Green Algae, and a Proposal to Reclassify Formosa maritima Cao et al. 2020 and Bizionia arctica Li et al. 2015 as Xanthomarina New Members
Source: Microorganisms. 2026 Jan 30;14(2):328. doi: 10.3390/microorganisms14020328 (PMC12943765; doi:10.3390/microorganisms14020328)

Genome analysis and characterization of *Formosa bonchosmolovskayae* sp. nov. isolated from brown and green algae, and a proposal to reclassify *Formosa maritima* Cao et al. 2020 and *Bizionia arctica* Li et al. 2015 as *Xanthomarina* new members

Olga Nedashkovskaya[1,*], Evgeniya Bystritskaya[1], Yulia Savicheva[1], Yulia Bronnikova [1], Nadezhda Otstavnykh[1], Viacheslav Eremeev[1], Song-Gun Kim[2], Natalia Zhukova[3], and Marina Isaeva[1,*]

[1] G.B. Elyakov Pacific Institute of Bioorganic Chemistry, Far Eastern Branch, Russian Academy of Sciences, Prospect 100 Let Vladivostoku, 159, Vladivostok 690022, Russia; oned2004@mail.ru (O.N.); ep.bystritskaya@yandex.ru (E.B.); molonova.yulya@gmail.com (Y.B.); chernysheva.nadezhda@gmail.com (N.O.); iu.savicheva0@yandex.ru (Y.S.) wieremeew@gmail.com (V.E.); issaeva@gmail.com (M.I.)

[2] Korean Collection for Type Cultures, Biological Resource Center, Korea Research Institute of Bioscience and Biotechnology, 181 Ipsin-gil, Jeongeup-si 56212, Republic of Korea; sgkim@kribb.re.kr (S-G.K.)

[3] A.V. Zhirmunsky National Scientific Center of Marine Biology, Far Eastern Branch, Russian Academy of Sciences, Palchevskogo Street 17, Vladivostok 690041, Russia; nzhukova35@list.ru (N.Z.)

* Correspondence: oned2004@mail.ru (O.N.); issaeva@gmail.com (M.I.); Tel.: +7-423-231-1168 (O.N.)

Supplementary Materials

Table S1. Genomic features of type strains of *Xanthomarina* genus, *F. maritima* 1494^T 4, and *B. arctica* CGMCC 1.12751^T.

Feature	1	2	3	4
Assembly level	Contig	Scaffold	Scaffold	Contig
Genome size (Mb)	3.1	3.2	3	3.9
Number of contigs	47	30	76	22
G+C Content (mol%)	35.5	31	31	33
N50 (Mb)	0.164	0.404	0.141	1.1
L50	5	3	8	2
Coverage (x)	690	325	644	340
Total genes	2,828	2,950	2,838	3,499
Protein coding genes	2,771	2,894	2,775	3,439
rRNAs (5S/16S/23S)	0/2/1	2/1/1	1/1/3	3/0/0
tRNA	51	36	36	38
checkM completeness (%)	100	99.68	99.35	99.68
checkM contamination (%)	0.49	0.32	0.16	0.32
WGS project	ANLA01	QGGP01	VSFC01	BMFQ01
Genome assembly name	Formosa sp. AK20	ASM314864v1	ASM808490v1	ASM1463891v1

Strains: **1**, *Xanthomarina gelatinilytica* AK20^T; **2**, *Xanthomarina spongicola* DSM 22637^T; 3, *F. maritima* 1494^T; 4, *B. arctica* CGMCC 1.12751^T.

Figure S1. Two-dimensional thin-layer chromatogram of polar lipids extracted from strain 4Alg 33^T (a), 3Alg 14/1 (b), *Formosa undariae* KCTC 32328^T (c), *Xanthomarina spongicola* KCTC 22662^T (d) and *Formosa maritima* KCTC 72531^T (e). PE, phosphatidylethanolamine; AL1-2, unidentified aminolipids, L1-4, unidentified lipids.

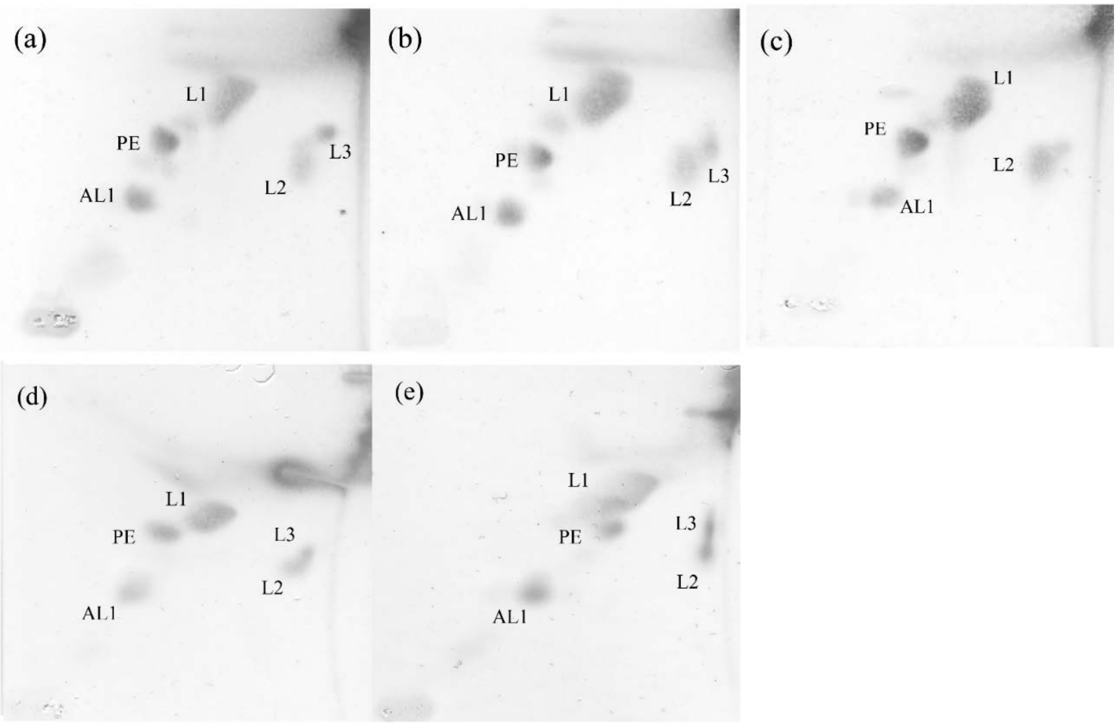

Figure S2: Two-dimensional thin-layer chromatogram of polar lipids extracted from strain *Formosa maritima* KCTC 72531^T (a) and *Xanthomarina spongicola* KCTC 22662^T (b). PE, phosphatidylethanolamine; AL1-2, unidentified aminolipids, L1-4, unidentified lipids.

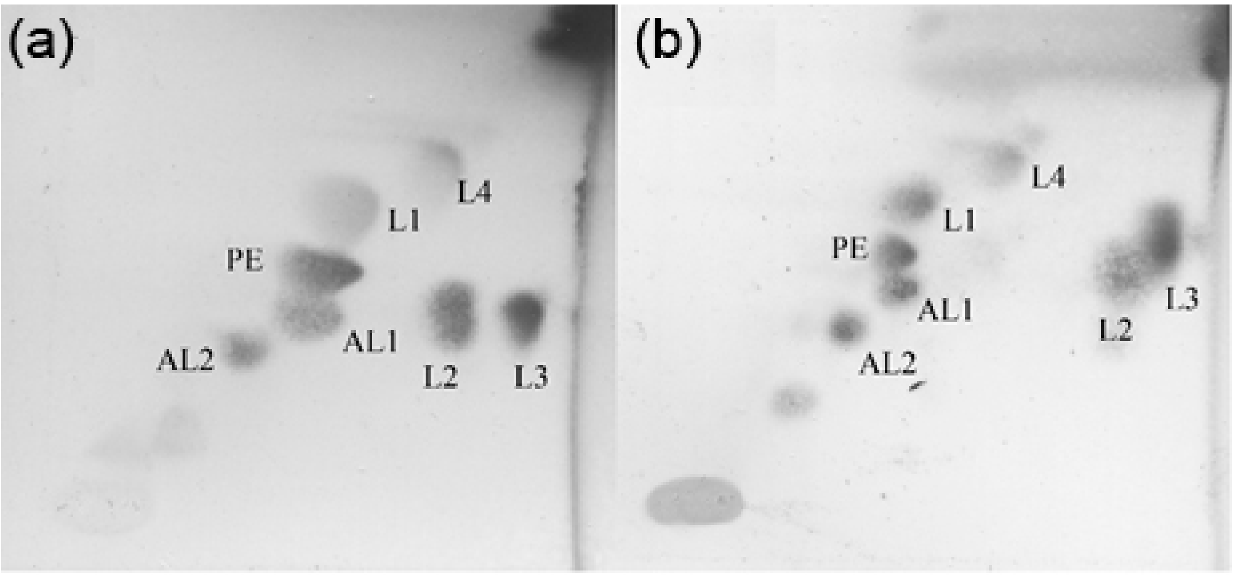

Supplement: Supplementary file 1 [file microorganisms-14-00328-s001.zip › microorganisms-4077872-supplementary.pdf]
